# Cellular overexpression of mutant TDP‐43 results in a decrease in solubility of phosphorylated TDP‐43

**DOI:** 10.1002/alz.090006

**Published:** 2025-01-03

**Authors:** Matthew B Dopler, Keyshawn E Cox, Tyler L Petersen, Muhammad I Abeer, Juneessa M Pressley, Sanaz Arezoumandan, Michael A. Gitcho

**Affiliations:** ^1^ Delaware State University, Dover, DE USA; ^2^ Delaware Center for Neuroscience Research, Dover, DE USA

## Abstract

**Background:**

Amyotrophic lateral sclerosis (ALS) is a progressive neurodegenerative disease that is characterized by upper and lower motor neuron death that leads to paralysis with the average survival being 3‐5 years after diagnosis. The major pathological protein in ALS is TDP‐43. TDP‐43 becomes hyperphosphorylated and forms inclusions mainly in the cytoplasm. Although only 5‐10% of familial cases are caused by mutations in TDP‐43; over 90% of all sporadic and familial ALS have TDP‐43 pathology. We have developed a cellular over expression model of TDP‐43 aggregation.

**Methods:**

Three familial ALS mutations (A315T, M337V, and S379P) were introduced in the *TARDBP* gene (3X‐TDP‐43) and cloned into pcDNA 3.1+ then transfected into HEK293 cells. Techniques used: Cell culture, Western blot, immunoprecipitation, immunofluorescence, nuclear/cytoplasmic fractionation, cellular solubility fractionation, and proteomics.

**Results:**

Overexpressing 3X‐TDP‐43 in HEK cells results in an increase in TDP‐43 C‐terminal fragments, time dependent change in TDP‐43 localization from nucleus to the cytoplasm, and an increase in cytoplasmic phosphorylated TDP‐43. We observed reduced solubility of phosphorylated TDP‐43 and a time dependent flux of LC3B and an increase in P62 which may indicate potential autophagy dysfunction. Global shotgun proteomics reveal changes in protein expression in importin subunit alpha‐1 (KPNA2), heat shock 70 kDa protein 1A (HSPA1A), H2B clustered histone 17 (H2BC17), and protein disulfide‐isomerase A3 (PDIA3). TDP‐43 also interacts in complex with KPNA2 and PDIA3.

**Conclusion:**

This cellular overexpression model shows a change in solubility and aggregation of phosphorylated TDP‐43. Hopefully this model will provide a better understanding of the pathogenesis of TDP‐43 proteinopathies

